# Supplementary material for: Time-resolved THz Stark spectroscopy of molecules in solution
Source: Nat Commun. 2024 May 17;15:4212. doi: 10.1038/s41467-024-48164-w (PMC11101612; doi:10.1038/s41467-024-48164-w)
Supplement: Supplementary file 1 — Supplementary Information [file 41467_2024_48164_MOESM1_ESM.pdf]

Supplementary Information: Time-resolved THz Stark spectroscopy of molecules in solution

Bong Joo Kang[1,5], Egmont J. Rohwer[1], David Rohrbach[1], Elnaz Zyaee[1], Maryam Akbarimoosavi[1], Zoltan Ollmann[1], Gleb Sorohhov[2], Alex Borgoo[3], Michele Cascella[3], Andrea Cannizzo[1], Silvio Decurtins[2], Robert J. Stanley[4], Shi-Xia Liu[2] and Thomas Feurer*[1]

[1]Institute of Applied Physics, University of Bern, Bern, 3012, Switzerland.
[2]Department of Chemistry, Biochemistry and Pharmaceutical Sciences, University of Bern, Bern, 3012, Switzerland.
[3]Department of Chemistry and Hylleraas Centre for Quantum Molecular Sciences, University of Oslo, Oslo, N-0315, Norway.
[4]Department of Chemistry, Temple University, Philadelphia, 19122, Pennsylvania, United States.
[5]Division of Advanced Materials, Korea Research Institute of Chemical Technology (KRICT), Daejeon, 34114, Republic of Korea.
* corresponding author: thomas.feurer@unibe.ch.

Table of Contents

1. Synthesis and preparation of molecular systems

2. Density Functional Theory calculations

3. Conventional Stark spectroscopy

4. THz Stark spectroscopy

5. Additional THz Stark spectroscopy results

6. Characterization of THz pulses

7. Liptay analysis

8. Local field correction factor

9. THz Stark spectroscopy results of anthanthrene in EtOAc

1 Synthesis and preparation of molecular systems

The two molecular systems were selected because they show either a pronounced change in dipole moment, which allows us to unambiguously observe the linear Stark effect, or a pronounced change in polarizability, which allows us to observe the quadratic Stark effect. In addition, both molecules are relevant in the field of molecular electronics and the extracted physicochemical properties are relevant in their own right.

The first molecular system is fused heterocyclic tetrathiafulvalene [1–5] – benzothiadiazole [6–8] (TTF-BTD) with Br substituted at the 4 and 8 positions of BTD [9]. This molecule is known to undergo intramolecular charge transfer [10, 11] with a correspondingly large change in dipole moment upon excitation of the HOMO-LUMO transition. The relatively broad absorption band centered around 500 nm is a typical signature of such intramolecular charge transfer.

The second molecular system is the 4,6,10,12-tetrakis(triisopropylsilylethynyl)-anthanthrene compound [12], which bears a graphene-like aromatic skeleton and is well-known for its quantum interference effect on the single molecule conductance, [13–15] and for its versatile chromophoric properties in organic electronics [16–20]. It exhibits optically allowed transitions with significant oscillator strengths in the spectral range around 500 nm.

For all experiments, toluene was used as solvent because it has a low polarity and forms a transparent optical glass at 77 K, which is mandatory for the conventional Stark measurements. The low polarity limits effects due to varying solvent polarity at different temperatures and the potential for THz field-induced orientation of the solvent. For conventional Stark measurements, the sample concentration was 1 mM for TTF-BTD and 0.45 mM for anthanthrene. The sample concentration for the THz Stark measurements was 1 mM for TTF-BTD and 0.5 mM for anthanthrene. In the THz Stark experiment, a spectrosil quartz flow cell from Starna Scientific was used with a sample thickness of 200 μm and a wall thickness of 200 μm. Even though a flow cell is not specifically needed, it helped to suppress air bubble formation. Furthermore, any potential effects due to toluene evaporation are reduced.

While the ground state absorption spectra in conventional Stark spectroscopy were measured directly with the Stark spectroscopy apparatus (with no applied electric field at low temperature), the room temperature ground state absorption spectra were recorded separately with a spectrophotometer (Perkin-Elmer Lambda 750 Spectrometer, 1 mm thick cuvette).

2 Density Functional Theory calculations

We performed DFT calculations of both systems to gain further insight into the charge redistribution between the molecular orbitals involved in the observed optical transitions and the associated energies. While details on our DFT computations on TTF-BTD have already been published elsewhere [21], we here present methods and results on the second system anthanthrene. The molecular geometries of anthanthrene were optimised at the Kohn-Sham DFT level. We used the PBE0 [22] or the B3LYP [23] functionals to approximate the exchange-correlation energy in combination with the 6-31+G(d,p) basis set [24]. The excitation energies were computed by time-dependent DFT (TD-DFT) [25]. The properties of excited states were obtained as higher-order response properties of the ground state. Specifically, the polarizability of an excited state was determined by first converging the electronic energy of the ground state and then by computing the double residue of the cubic response function as described in reference [26] and implemented in the DALTON software package [27]. Table S1 summarizes possible transitions originating from the ground S_0 state with corresponding wavelength, oscillator strength, major molecular orbital contributions, and transition dipole moment. Molecular orbitals and associated energies involved in the calculated transitions for the sample are illustrated in Fig. S1.

Table S1 The ground state transitions of anthanthrene with wavelength, oscillator strength, and major contributing molecular orbitals calculated with TD-DFT.

Excited State	Wavelength (nm)	Oscillator strength	Major contributions (%)	
**S₁**	**500.2**	**0.4846**	**HOMO → LUMO Pure**	
S_2	419.7	0.0	HOMO-1→LUMO<br>HOMO→LUMO+1	36<br>13
S_3	402.8	0.0	HOMO-1→LUMO<br>HOMO→LUMO+1	13<br>36
S_4	395.2	0.0087	HOMO-2→LUMO<br>HOMO→LUMO+2	27<br>22
S_5	333.0	0.6133	HOMO-3→LUMO<br>HOMO-2→LUMO<br>HOMO-1→LUMO+1<br>HOMO→LUMO+2	3<br>20<br>2<br>22
S_6	315.4	0.0366	HOMO-3→LUMO<br>HOMO→LUMO+2<br>HOMO→LUMO+3	33<br>2<br>12

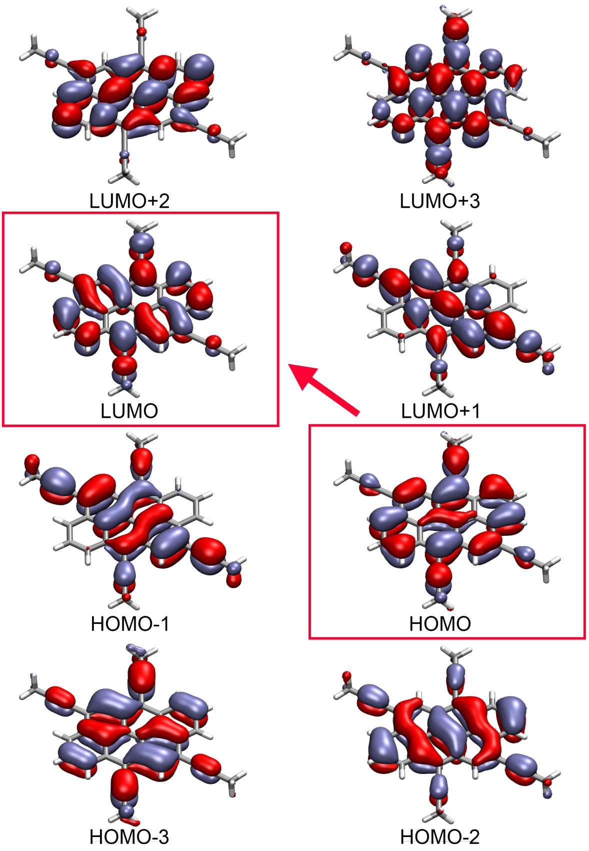

Fig. S1 Molecular orbitals of anthanthrene. With reference to the main text, we draw the reader's attention to the HOMO→LUMO transition in particular.

Table S2 $\Delta\alpha$ computed by TD-DFT with an aug-cc-PVDZ basis set.

State	Wavelength (PBE0)	Wavelength (B3LYP)	Tr($\Delta\alpha$) (B3LYP)
S_1	500.2 nm	518.8 nm	457 $Å^3$
S_5	333.0 nm	347.0 nm	333 $Å^3$

The average isotropic change in polarizability over the transitions was calculated using both PBE0 and B3LYP functionals. We checked convergence with the basis set (using aug-cc-PVTZ and daug-cc-PDVZ). We verified that using aug-cc-PVDZ the basis set error affects the quantitative result by less than 1%. In addition, note that the lowest energy transition $S_0 \rightarrow S_1$ splits in a well-separated vibronic progression.

3 Conventional Stark spectroscopy

To understand the limitations of conventional field Stark spectroscopy we will describe the technique in general terms here. A more detailed description of the specific Stark spectrometer used, along with technical details can be found in a previous publication [21]. Typically, a high voltage source is connected to transparent electrodes (indium tin oxide: ITO) on the inner front and back surface of the sample cell. The front and the back window are separated by a Kapton spacer with a thickness of 25 µm and the sample cavity is filled by injection with the sample solution. The sample is then mounted in a cooled Dewar at 77 K to freeze the solvent. Freezing the sample is also a common work-around to increase the breakdown voltage and prevent unwanted redox chemistry. During the experiments, a 3.5 kHz sinusoidal signal is applied across a cell. While for TTF-BTD the applied voltage was $V_{\mathrm{rms}} = 250\,\mathrm{V}$ (with a peak field of 141 kV/cm), for anthanthrene the applied voltage was $V_{\mathrm{rms}} = 150\,\mathrm{V}$ (with a peak field of 85 kV/cm). A lamp and monochromator provide tunable probe light. The electric field-induced transmission changes are monitored at twice the AC frequency by a lock-in amplifier. The difference between the in- and out-of-phase components is plotted as a function of monochromator wavelength, resulting in a Stark spectrum. The cell can be rotated about a vertical axis within the Dewar to facilitate different relative angles between the applied field and the probe light polarization. The polarization of the probe light is set with a Glan-Taylor prism.

4 THz Stark spectroscopy

This section describes in detail all relevant steps to extract the molecular physicochemical constants. We start with a brief description of the experimental apparatus and the data recorded by it. Next, we outline how we correct for the group velocity dispersion of the probe pulses and give a detailed account of the background subtraction protocol. A further important ingredient for the analysis is the THz electric field strength in the sample and we determine it by a combination of measurements and finite difference time domain simulations. Finally, the measurements are analyzed by the Liptay formalism, which is briefly described toward the end.

Experimental setup

Figure S2 shows a schematic of the experimental setup. The high-field single-cycle THz waveforms were generated via tilted-pulse-front pumping in $LiNbO_3$ and the time-delayed femtosecond supercontinuum (fs-SC) probe pulses came from white-light generation in a 5-mm-thick CaF_2 crystal, which was mounted in a continuously moving mount in order to avoid photo-darkening. In detail, a 1 kHz Ti:sapphire regenerative amplifier (Legend Elite Duo Femto, Coherent) delivering 90 fs pulses with an average power of 8 W at 800 nm was used to produce the single-cycle THz waveforms by optical rectification in a prism-cut $LiNbO_3$ crystal [28, 29]. The THz waveforms were imaged to the sample position by a combination of two lenses with focal lengths of 100 mm and 50 mm, resulting in a 2:1 demagnification.

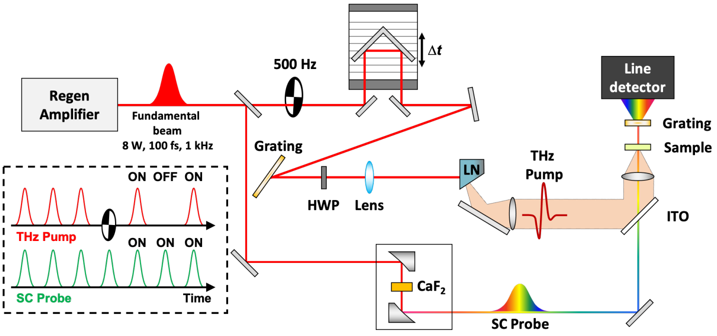

Fig. S2 THz Stark spectroscopy. High-field single-cycle THz waveforms were generated via tilted-pulse-front pumping in $LiNbO_3$ and the fs-SC probe pulses were generated in CaF_2. The probe pulses passed collinearly with the THz pulses through the sample and were analyzed by a spectrometer. A chopper running at 500 Hz alternated between THz waveform on and off.

THz waveform and fs-SC probe pulses were combined collinearly with an indium tin oxide (ITO) coated glass slide, which acts as a mirror at THz frequencies but is transparent at optical frequencies. The relative polarization

between the THz waveform and the probe pulse was adjusted by an achromatic half-wave plate in the probe arm and both were subsequently focused to the sample using a TPX lens. The probe beam waist was set to $w_{\mathrm{SC}} \approx 17\ \mu$m, which is more than one order of magnitude smaller than the THz beam waist of $w_{\mathrm{THz}} \approx 1$ mm, and thus probes an area of almost constant THz electric field strength at the center of the THz spot. After the sample, the probe spectrum was analyzed with a 1024-pixel CMOS array (Glaz-I, Synertronic Designs) on a pulse-to-pulse basis. A phase-locked chopper blocked every other THz waveform and from two consecutive probe spectra (with and without THz) the change in absorption, i.e. $\Delta A(\lambda) = A_{\mathrm{THz\ on}}(\lambda) - A_{\mathrm{THz\ off}}(\lambda) = -\log_{10}(T_{\mathrm{THz\ on}}(\lambda)/T_{\mathrm{THz\ off}}(\lambda))$, was calculated as a function of wavelength λ. In order to realize a sufficiently high signal-to-noise ratio we typically averaged more than 5000 pulse pairs. From a number of such measurements for different time delays τ between the THz waveform and the probe pulse, we construct two-dimensional color-coded Stark maps $\Delta A(\tau, \lambda)$ as shown in Fig. S3a.

Group velocity dispersion correction

The fs-SC probe pulses experience group velocity dispersion (GVD) due to a number of dispersive optical elements through which they have to propagate. As a result, each spectral component has a different effective time delay with respect to the THz waveform. Since the total GVD is independent of sample and polarization, it can be corrected for. As an example, Fig. S3a shows the measured and color-coded Stark signals (anthanthrene) versus time delay between probe pulse and THz waveform and wavelength of probe pulse. The black solid curve shows the 3rd order polynomial fitting, which is subsequently used for GVD correction. The GVD correction was confirmed by measuring the cross-phase modulation in a quartz substrate at the sample position. In essence, all rows of data are shifted by a time delay that is given by the 3rd order polynomial curve and Fig. S3b shows the data after GVD correction. All measurements on the solid curve shown in Fig. S3a now have the same corrected time delay.

Background measurements

To characterize the measurement background, we recorded signals for the pure solvent. Figure S4 shows the spectra for parallel and perpendicular orientation of THz and probe polarization. For all time delays, the measurement is dominated by random noise. Note that the GVD correction introduces an apparent parabolic structure. Around time delay zero we find a positive signal for all wavelength, which is attributed to a THz Kerr effect observed in low-polar liquids [30]. In Fig. S4c,d the two signals integrated along the wavelength axis are shown as function of time delay. In agreement with theory, the signal for parallel orientation is larger than that for perpendicular orientation. For all

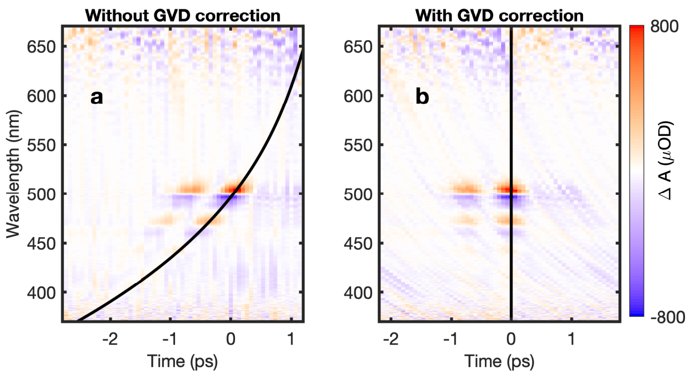

Fig. S3 Measured color-coded Stark map for anthanthrene versus time delay and wavelength before **a** and after **b** GVD correction. The black solid curve indicates the fitted 3rd order polynomial representing the GVD, which turns into a vertical line after GVD correction.

measurements presented in this paper, we subtracted this background signal before plotting and analysing the data.

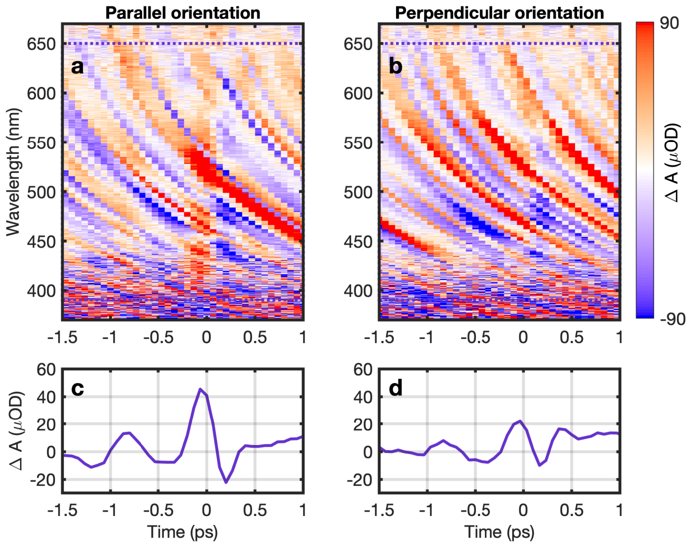

Fig. S4 Background measurement with pure toluene for **a** parallel and **b** perpendicular orientation of THz and probe pulses. Spectral average of the change in absorption between the purple dotted lines as a function of time delay for **c** parallel and **d** perpendicular orientation.

5 Additional THz Stark spectroscopy results

In the main text we only show results for parallel polarization between THz waveform and probe pulse. However, the Liptay analysis requires Stark signals for two different relative polarization orientations, ideally but not necessarily parallel and perpendicular. Hence, for completeness we here show the signals for perpendicular orientation. While Fig. S5 shows the perpendicular orientation for TTF-BTD, Fig. S6 shows the corresponding results for anthanthrene.

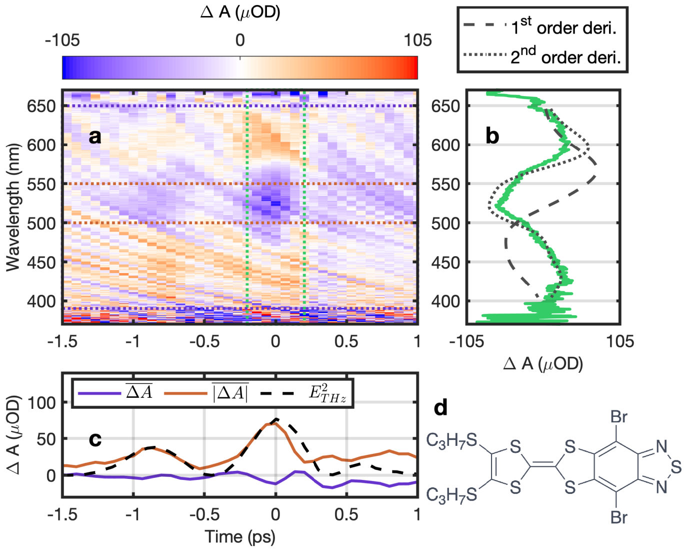

Fig. S5 THz Stark signal of TTF-BTD with perpendicular orientation. a False-color plot of the measured change in absorption spectrum as a function of time delay between THz and probe pulse and wavelength. **b** Time-averaged (between the two green dotted lines) change in absorption versus wavelength (green solid curve) compared to the scaled first (black dashed curve) and second order derivative (black dotted curve) of the ground state absorption spectrum. **c** Spectral average of the change in absorption between the purple dotted (purple curve) and red dotted lines (red curve) in **a**. The red curve is compared to the scaled square of the measured THz electric field E^2_{THz} (black dashed curve). **d** Chemical structure of TTF-BTD.

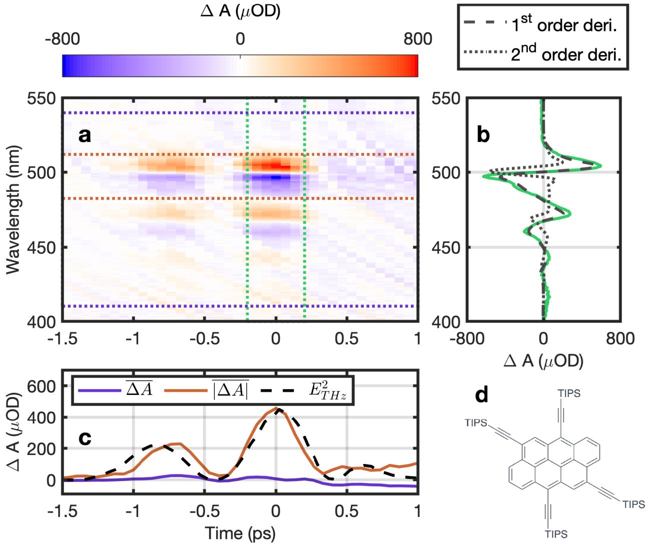

Fig. S6 THz Stark signal of anthanthrene with perpendicular orientation. a False-color plot of the measured change in absorption spectrum as a function of time delay between THz and probe pulse and wavelength. **b** Time-averaged (between the two green dotted lines) change in absorption versus wavelength (green solid curve) compared to the scaled first (black dashed curve) and second order derivative (black dotted curve) of the ground state absorption spectrum. **c** Spectral average of the change in absorption between the purple dotted (purple curve) and red dotted lines (red curve) in **a**. The red curve is compared to the scaled square of the measured THz electric field E^2_{THz} (black dashed curve). **d** Chemical structure of anthanthrene.

6 Characterization of THz pulses

To determine the THz electric field strength in air we assume that the spatio-temporal electric field can be expressed in a product $E(x, y, t) = E_0 g_x(x) g_y(y) f(t)$, where E_0 is the peak electric field strength and $g_x(x)$, $g_y(y)$, and $f(t)$ are spatially and temporally dependent functions normalized to a peak value of one. The peak electric field strength E_0 was determined from three measurements, i.e. the average power P_{avg} at a repetition rate of f_{rep}, the spatial profiles $g_x^2(x)$ and $g_y^2(y)$, and the time dependence $f(t)$ and is calculated via

$$E_0 = \sqrt{\frac{P_{avg}}{\epsilon_0 c f_{rep} \int_{-\infty}^{\infty} g_x^2(x)dx \int_{-\infty}^{\infty} g_y^2(y)dy \int_{-\infty}^{\infty} f^2(t)dt}}, \quad (1)$$

where ϵ_0 is the vacuum permittivity and c is the speed of light in vacuum. The average power was recorded by a calibrated THz power meter (with a resolution of 50 µW and a relative error of ±12%), the spatial profiles were extracted from two perpendicular knife-edge measurements, and the time dependence was measured by electro-optic sampling in a 0.3-mm-thick GaP $< 110 >$ crystal. Note that all measurements are taken at the sample position. The maximum THz power was measured to 3.6 mW at 1 kHz repetition rate, the THz spot was nearly Gaussian with a beam waist of 1 mm in x- as well as in y-direction, and the measured electro-optic signal [31–33] and the corresponding spectrum are shown in Fig. S7a and b. Inserting the three measurements in Eq.(1) results in a peak electric field of $E_0 = (400 \pm 24)$ kV/cm in air. The corresponding THz spectral amplitude shows a center frequency of 0.5 THz with a bandwidth of 0.7 THz (FWHM). The optical system was in ambient atmosphere, hence the absorption feature around 1.1 THz is due to the water vapor absorption.

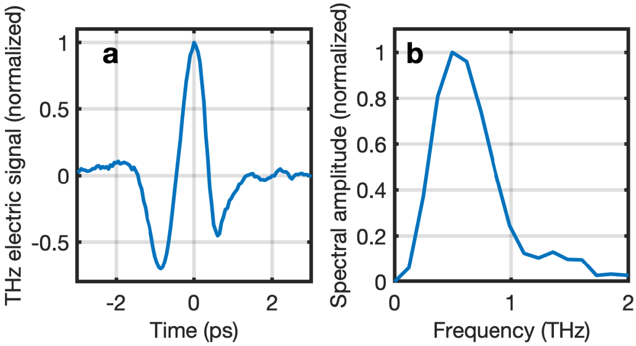

Fig. S7 **a** Measured electro-optic signal at the sample position and **b** corresponding spectrum.

The effective field strength, at which the probe pulse interrogates the molecular system, is typically smaller because of a number of effects. The dominating reduction factor comes from the geometry and material of the cuvette. For instance, Fresnel reflections or Fabry-Perot interferences at or within the cuvette limit the maximum electric field strength. Some solvents also have a no-negligible THz absorption coefficient and consequently reduce the electric field strength exponentially along the probe's propagation direction. Moreover, several effects lead to a smearing and averaging of the signal, such as the finite size of the probe interrogating the sample at different THz electric field strengths or the finite duration of the probe pulse and the group velocity mismatch between THz waveform and probe pulse. In order to account for all these effects we performed finite difference time-domain simulations propagating the THz waveform together with a time delayed probe pulse through the sample cuvette. We found that the time dependence of the THz electric field experienced by the probe pulse is almost identical to the free space THz waveform, however the peak electric field strength is reduced by a factor of 0.7 resulting in a maximum electric field of $E_0 = (280 \pm 17)\,\mathrm{kV/cm}$ in the sample.

7 Liptay analysis

The following analysis closely follows the recipe outlined in reference [34]. After having identified the Stark-active transitions, the Stark spectra are subsequently analyzed with the Liptay formalism (for details also see references [21] and [35]). The analytic expression derived by Liptay links the molar absorption $\Delta\epsilon(\bar{\nu})$ as a function of wavenumber to the square of the electric field $\mathbf{E}$ and ground state absorption spectrum $\epsilon(\bar{\nu})$

$$\Delta\epsilon(\bar{\nu}) = f_l^2|\mathbf{E}|^2 \left\{ A_\chi \epsilon(\bar{\nu}) + \frac{B_\chi}{15hc}\bar{\nu}\frac{\mathrm{d}}{\mathrm{d}\bar{\nu}}\left(\frac{\epsilon(\bar{\nu})}{\bar{\nu}}\right) + \frac{C_\chi}{30h^2c^2}\bar{\nu}\frac{\mathrm{d}^2}{\mathrm{d}\bar{\nu}^2}\left(\frac{\epsilon(\bar{\nu})}{\bar{\nu}}\right)\right\}, \quad (2)$$

where h is Planck's constant and c the speed of light. The model assumes an isotropic distribution of transition dipole moments, which is provided by freezing the sample in conventional Stark spectroscopy. The measured Stark spectra are fitted with a weighted combination of the zeroth, first, and second order derivative of the ground state absorption spectrum. The coefficient A_χ is determined by the transition polarizability and/or the transition hyperpolarizability of the sample, which can usually be neglected for immobilized samples. The second and third coefficients are given by

$$B_\chi = \frac{5}{2}\mathrm{Tr}(\underline{\Delta\alpha}) + \left(3\cos^2\chi - 1\right)\left(\frac{3}{2}\mathbf{m}\underline{\Delta\alpha}\mathbf{m} - \frac{1}{2}\mathrm{Tr}(\underline{\Delta\alpha})\right) \quad (3)$$

$$C_\chi = |\Delta\mu|^2\left\{5 + \left(3\cos^2\chi - 1\right)\left(3\cos^2\zeta - 1\right)\right\}, \quad (4)$$

where $\mathrm{Tr}(\underline{\Delta\alpha})$ is the trace of the polarizability tensor, $\mathbf{m}\underline{\Delta\alpha}\mathbf{m}$ is its projection along the transition dipole moment, χ is the angle between the applied electric field and the probe polarization, $\Delta\mu$ is the change in dipole moment and ζ is the angle between the change in dipole moment and the transition dipole moment.

The molecular parameters are extracted by simultaneously fitting the ground-state absorption spectra and two Stark spectra for different probe polarizations. Figure S8 shows the measured data (dotted curves) and the corresponding fits (black solid curves) for the TTF-BTD sample. Figure S8a and Fig. S8c show the results for the conventional Stark measurement at 77 K, while Fig. S8b and Fig. S8d show the results for the THz-Stark measurement at 295 K (same data as shown in Fig. 4 in the main text). For both experiments we find reasonable agreement between the fits and the measured data with a slightly better fit quality for the conventional Stark measurements. Figure S8e and Fig. S8f separately show the contribution of the zeroth, first and second order contribution to the fitted Stark signal for $\chi = 90°$. The contribution of the zeroth order $\widehat{A}_\chi$ is multiplied by 10 and we a find negligible contribution to the Stark signal for both the conventional and the THz experiment, which confirms that the alignment of molecules due to the electric field is negligible.

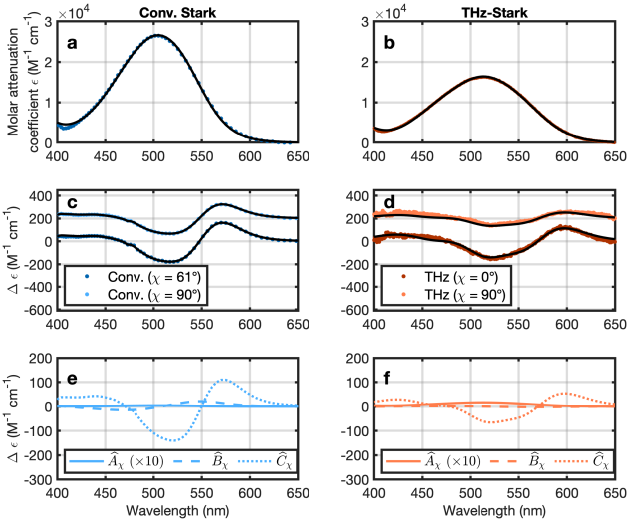

Fig. S8 TTF-BTD conventional Stark and THz-Stark spectra fitting a, b Ground-state absorption spectrum ϵ of TTF-BTD sample at 77 K (**a**) and at 295 K (**b**). The dots represent the data points and the black solid curves represents the fits. **c, d** Measured Stark spectra for two different incidence angles (dots) and corresponding fits (black solid curves) for the conventional Stark measurement **c** and THz-Stark measurement **d**. For better visualization, the curves for $\chi = 90°$ are arbitrarily shifted along the $\Delta\epsilon$-axis. **e, f** Contribution of the zeroth (solid curve), first (dashed curve) and second (dotted curve) order derivative line form for the Stark spectra for $\chi = 90°$.

The same fitting results are shown in Fig. S9 for the anthanthrene sample. Also here we find reasonable agreement between the fits and the measured data for both conventional and THz Stark spectroscopy.

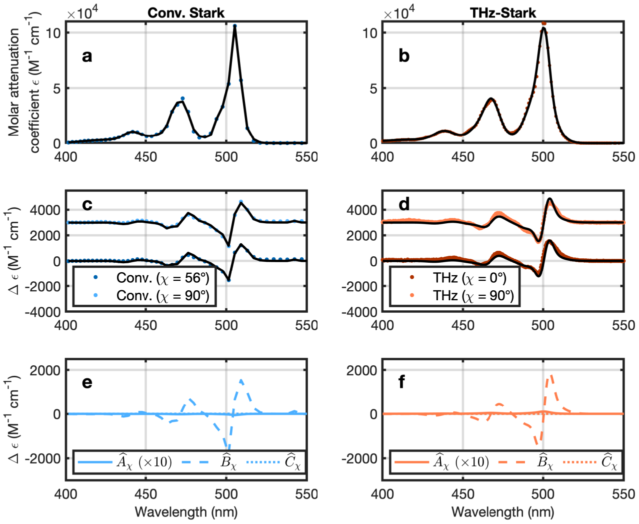

Fig. S9 Anthanthrene conventional Stark and THz-Stark spectra fitting a, b Ground-state absorption spectrum ϵ of anthanthrene sample at 77 K (**a**) and at 295 K (**b**). The dots represent the data points and the black solid curves represents the fits. **c, d** Measured Stark spectra for two different incidence angles (dots) and corresponding fits (black solid curves) for the conventional Stark measurement **c** and THz-Stark measurement **d**. For better visualization, the curves for $\chi = 90°$ are arbitrarily shifted along the $\Delta\epsilon$-axis. **e, f** Contribution of the zeroth (solid curve), first (dashed curve) and second (dotted curve) order derivative line form for the Stark spectra for $\chi = 90°$.

8 Local field correction factor

The local field correction factor gives a measure of how the solvent cavity affects the field inside the cavity when an external electric field is applied. The calculations were done in analogy to those described in literature [36–38]. We approximated the molecule as occupying a cavity with an ellipsoidal shape. For anthanthrene we estimate the ellipsoid axes to 15 Å, 15 Å, and 3 Å, while for the TTB-BTD we estimate them to 15 Å, 7 Å, and 3 Å. Note that reasonable variation of these parameters has only minor effects to the local field correction factor. Based on literature, the dielectric constant of toluene at 77 K and zero frequency is 2.52 [39], while at room temperature and 400 GHz it is 2.27 [40]. The local field correction factors f_l for TTF-BTD and anthanthrene are estimated to be 1.30 and 1.33 respectively for conventional Stark spectroscopy and to be 1.26 and 1.29 respectively for THz Stark spectroscopy. Hence, the fitted values $\mathrm{Tr}(\Delta\alpha)$ have to be divided by ${f_l}^2$ and $\Delta\mu$ by f_l before the numbers can be compared with the DFT calculation.

9 THz Stark spectroscopy results of anthanthrene in EtOAc

To demonstrate that the transient THz Stark effect can be studied in a different solvent with different polarity, we measured the same solute Anthanthrene in the solvent ethyl acetate (EtOAc). The relative polarity of EtOAc is about 0.228 and the relative polarity of toluene is about 0.099. To apply Liptay analysis, Stark signals were measured for two different relative polarization orientations. While Fig. S10 shows the parallel orientation for Anthanthrene in EtOAc, Fig. S11 shows the perpendicular orientation for Anthanthrene in EtOAc. Although the relative polarity increased by a factor of 2.3, the result of EtOAc gives a similar Stark signature to those measured for solutes in toluene as shown in Fig. S12. The blue shift in the ground state absorption is attributed to the solvent's instantaneous electronic polarizability. Note that the solute we used is a non-polar solute and cannot be dissolved in more polar solvents.

Then, we compared quantitatively the extracted molecular parameters as calculated via DFT or measured by conventional and THz Stark spectroscopy summarized in Table S3.

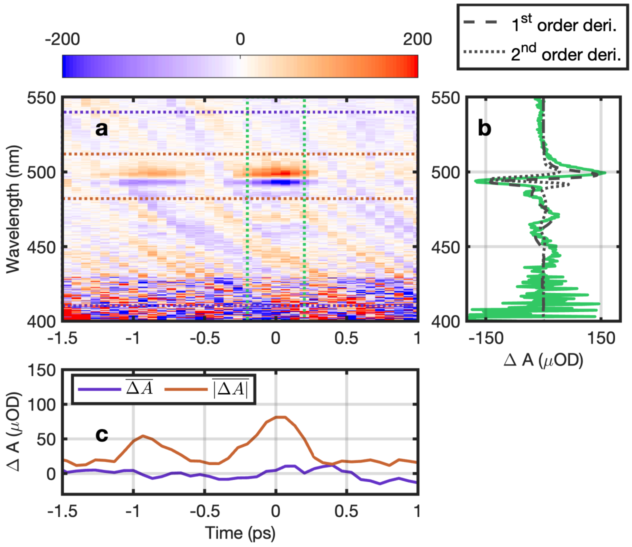

Fig. S10 THz Stark signal of anthanthrene in EtOAc with parallel orientation. **a** False-color plot of the measured change in absorption spectrum as a function of time delay between THz and probe pulse and wavelength. **b** Time-averaged (between the two green dotted lines) change in absorption versus wavelength (green solid curve) compared to the scaled first (black dashed curve) and second order derivative (black dotted curve) of the ground state absorption spectrum. **c** Spectral average of the change in absorption between the purple dotted (purple curve) and red dotted lines (red curve) in **a**.

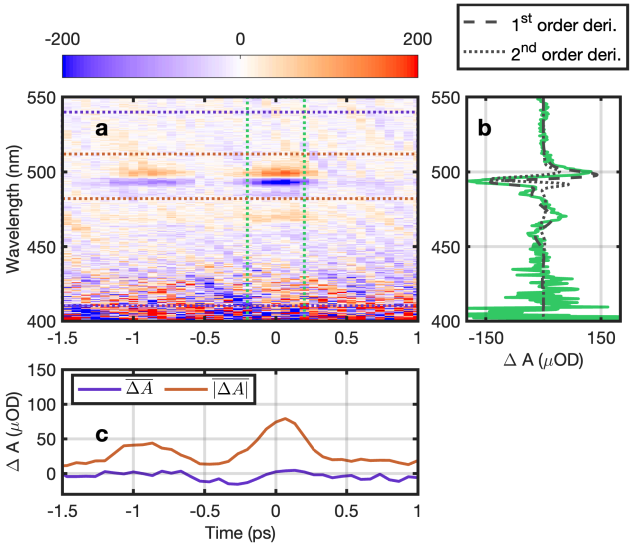

Fig. S11 THz Stark signal of anthanthrene in EtOAc with perpendicular orientation. a False-color plot of the measured change in absorption spectrum as a function of time delay between THz and probe pulse and wavelength. **b** Time-averaged (between the two green dotted lines) change in absorption versus wavelength (green solid curve) compared to the scaled first (black dashed curve) and second order derivative (black dotted curve) of the ground state absorption spectrum. **c** Spectral average of the change in absorption between the purple dotted (purple curve) and red dotted lines (red curve) in **a**.

Table S3 Comparison of relevant molecular parameters as calculated via DFT or measured by conventional and THz Stark spectroscopy.

Parameter	DFT	Conventional Stark (Toluene)	THz-Stark (Toluene)	THz-Stark (EtOAc)
anthanthrene: 4,6,10,12-tetrakis(triisopropylsilylethynyl)anthanthrene				
$\mathrm{Tr}(\Delta\alpha)$ (Å^3)	457	363 ± 20	296 ± 70	229 ± 53

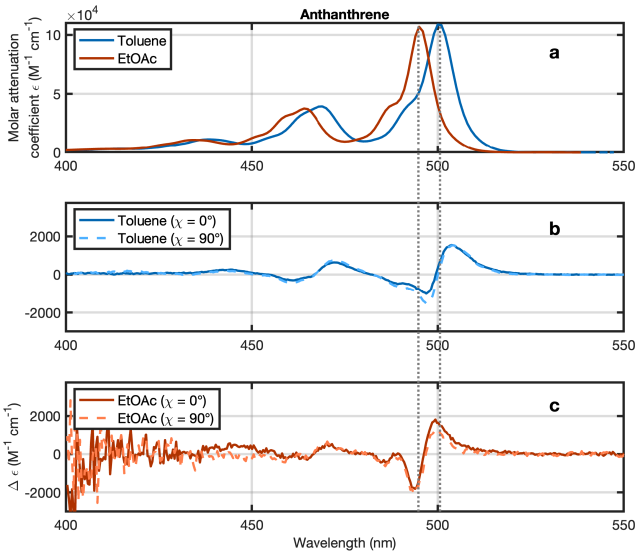

Fig. S12 Comparison of THz Stark signal of Anthanthrene in toluene and EtOAc. **a** absorption spectra of Anthanthrene in toluene (blue) and EtOAc (red). **b,c** THz Stark spectra of Anthanthrene (b) in toluene (c) in EtOAc recorded for parallel and perpendicular orientation of THz and probe polarization. For direct comparison the y-scale is in units of $\Delta\epsilon$ scaled to an electric field of 1 MV/cm. The grey dotted vertical lines are guides to the eye and help to visualize the shift of the spectra at the different temperatures.

References

[1] Bendikov, M., Wudl, F., Perepichka, D.F.: Tetrathiafulvalenes, Oligoacenenes, and Their Buckminsterfullerene Derivatives: The Brick and Mortar of Organic Electronics. Chem. Rev. **104**(11), 4891–4946 (2004). https://doi.org/10.1021/cr030666m

[2] Martín, N.: Tetrathiafulvalene: the advent of organic metals. Chem. Commun. **49**(63), 7025–7027 (2013). https://doi.org/10.1039/C3CC00240C

[3] Wu, J., Dupont, N., Liu, S.-X., Neels, A., Hauser, A., Decurtins, S.: Imidazole-annulated tetrathiafulvalenes exhibiting pH-tuneable intramolecular charge transfer and redox properties. Chem. Asian J. **4**(3), 392–399 (2009). https://doi.org/10.1002/asia.200800322

[4] Bergkamp, J.J., Decurtins, S., Liu, S.-X.: Current advances in fused tetrathiafulvalene donor–acceptor systems. Chem. Soc. Rev. **44**(4), 863–874 (2015). https://doi.org/10.1039/C4CS00255E

[5] Segura, J.L., Martín, N.: New Concepts in Tetrathiafulvalene Chemistry. Angew. Chem. Int. Ed. Engl. **40**(8), 1372–1409 (2001). https://doi.org/10.1002/1521-3773(20010417)40:8⟨1372::aid-anie1372⟩3.0.co;2-i

[6] Justin Thomas, K.R., Lin, J.T., Velusamy, M., Tao, Y.T., Chuen, C.H.: Color Tuning in Benzo[1,2,5]thiadiazole-Based Small Molecules by Amino Conjugation/Deconjugation: Bright Red-Light-Emitting Diodes. Adv. Funct. Mater. **14**(1), 83–90 (2004). https://doi.org/10.1002/adfm.200304486

[7] Wu, Y., Zhu, W.: Organic sensitizers from D–π–A to D–A–π–A: effect of the internal electron-withdrawing units on molecular absorption, energy levels and photovoltaic performances. Chem. Soc. Rev. **42**(5), 2039–2058 (2013). https://doi.org/10.1039/C2CS35346F

[8] Belton, C.R., Kanibolotsky, A.L., Kirkpatrick, J., Orofino, C., Elmasly, S.E.T., Stavrinou, P.N., Skabara, P.J., Bradley, D.D.C.: Location, Location, Location - Strategic Positioning of 2,1,3-Benzothiadiazole Units within Trigonal Quaterfluorene-Truxene Star-Shaped Structures. Adv. Funct. Mater. **23**(22), 2792–2804 (2013). https://doi.org/10.1002/adfm.201202644

[9] Pop, F., Riobé, F., Seifert, S., Cauchy, T., Ding, J., Dupont, N., Hauser, A., Koch, M., Avarvari, N.: Tetrathiafulvalene-1,3,5-triazines as (Multi)Donor–Acceptor Systems with Tunable Charge Transfer: Structural, Photophysical, and Theoretical Investigations. Inorg. Chem. **52**(9), 5023–5034 (2013). https://doi.org/10.1021/ic3027336

[10] Alemany, P., Canadell, E., Geng, Y., Hauser, J., Macchi, P., Krämer, K., Decurtins, S., Liu, S.-X.: Exploring the Electronic Structure of an Organic Semiconductor Based on a Compactly Fused Electron Donor–Acceptor Molecule. ChemPhysChem **16**(7), 1361–1365 (2015). https://doi.org/10.1002/cphc.201500090

[11] Geng, Y., Pfattner, R., Campos, A., Hauser, J., Laukhin, V., Puigdollers, J., Veciana, J., Mas-Torrent, M., Rovira, C., Decurtins, S., Liu, S.-X.: A Compact Tetrathiafulvalene–Benzothiadiazole Dyad and Its Highly Symmetrical Charge-Transfer Salt: Ordered Donor π-Stacks Closely Bound to Their Acceptors. Chem. – A Eur. J. **20**(23), 7136–7143 (2014). https://doi.org/10.1002/chem.201304688

[12] Giguère, J.-B., Verolet, Q., Morin, J.-F.: 4,10-Dibromoanthanthrone as a New Building Block for p-Type, n-Type, and Ambipolar π-Conjugated Materials. Chem. – A Eur. J. **19**(1), 372–381 (2013). https://doi.org/10.1002/chem.201202878

[13] Lambert, C.J., Liu, S.-X.: A Magic Ratio Rule for Beginners: A Chemist's Guide to Quantum Interference in Molecules. Chem. – A Eur. J. **24**(17), 4193–4201 (2018). https://doi.org/10.1002/chem.201704488

[14] Geng, Y., Sangtarash, S., Huang, C., Sadeghi, H., Fu, Y., Hong, W., Wandlowski, T., Decurtins, S., Lambert, C.J., Liu, S.-X.: Magic Ratios for Connectivity-Driven Electrical Conductance of Graphene-like Molecules. J. Am. Chem. Soc. **137**(13), 4469–4476 (2015). https://doi.org/10.1021/jacs.5b00335

[15] Famili, M., Jia, C., Liu, X., Wang, P., Grace, I.M., Guo, J., Liu, Y., Feng, Z., Wang, Y., Zhao, Z., Decurtins, S., Häner, R., Huang, Y., Liu, S.-X., Lambert, C.J., Duan, X.: Self-Assembled Molecular-Electronic Films Controlled by Room Temperature Quantum Interference. Chem **5**(2), 474–484 (2019). https://doi.org/10.1016/j.chempr.2018.12.008

[16] Geng, Y., Yi, C., Bircher, M.P., Decurtins, S., Cascella, M., Grätzel, M., Liu, S.-X.: Anthanthrene dye-sensitized solar cells: influence of the number of anchoring groups and substitution motif. RSC Adv. **5**(119), 98643–98652 (2015). https://doi.org/10.1039/C5RA21917E

[17] Giguère, J.-B., Sariciftci, N.S., Morin, J.-F.: Polycyclic anthanthrene small molecules: semiconductors for organic field-effect transistors and solar cells applications. J. Mater. Chem. C **3**(3), 601–606 (2015). https://doi.org/10.1039/C4TC02137A

[18] Zhang, L., Walker, B., Liu, F., Colella, N.S., Mannsfeld, S.C.B., Watkins, J.J., Nguyen, T.-Q., Briseno, A.L.: Triisopropylsilylethynyl-functionalized dibenzo[def,mno]chrysene: a solution-processed small molecule for bulk

heterojunction solar cells. J. Mater. Chem. **22**(10), 4266–4268 (2012). https://doi.org/10.1039/C2JM14998B

[19] Shah, B.K., Neckers, D.C., Shi, J., Forsythe, E.W., Morton, D.: Anthanthrene Derivatives as Blue Emitting Materials for Organic Light-Emitting Diode Applications. Chem. Mater. **18**(3), 603–608 (2006). https://doi.org/10.1021/cm052188x

[20] Shah, B.K., Neckers, D.C., Shi, J., Forsythe, E.W., Morton, D.: Photophysical Properties of Anthanthrene-Based Tunable Blue Emitters. J. Phys. Chem. A **109**(34), 7677–7681 (2005). https://doi.org/10.1021/jp052337z

[21] Rohwer, E.J., Akbarimoosavi, M., Meckel, S.E., Liu, X., Geng, Y., Lawson Daku, L.M., Hauser, A., Cannizzo, A., Decurtins, S., Stanley, R.J., Liu, S.-X., Feurer, T.: Dipole Moment and Polarizability of Tunable Intramolecular Charge Transfer States in Heterocyclic π-Conjugated Molecular Dyads Determined by Computational and Stark Spectroscopic Study. J. Phys. Chem. C **122**(17), 9346–9355 (2018). https://doi.org/10.1021/acs.jpcc.8b02268

[22] Adamo, C., Barone, V.: Toward reliable density functional methods without adjustable parameters: The PBE0 model. J. Chem. Phys. **110**(13), 6158–6170 (1999). https://doi.org/10.1063/1.478522

[23] Becke, A.D.: Density-functional thermochemistry. III. The role of exact exchange. J. Chem. Phys. **98**(7), 5648–5652 (1993). https://doi.org/10.1063/1.464913

[24] Hehre, W.J., Ditchfield, R., Pople, J.A.: Self—Consistent Molecular Orbital Methods. XII. Further Extensions of Gaussian—Type Basis Sets for Use in Molecular Orbital Studies of Organic Molecules. J. Chem. Phys. **56**(5), 2257–2261 (1972). https://doi.org/10.1063/1.1677527

[25] Runge, E., Gross, E.K.U.: Density-Functional Theory for Time-Dependent Systems. Phys. Rev. Lett. **52**(12), 997–1000 (1984). https://doi.org/10.1103/PhysRevLett.52.997

[26] Jansik, B., Jonsson, D., Sałek, P., Ågren, H.: Calculations of static and dynamic polarizabilities of excited states by means of density functional theory. J. Chem. Phys. **121**(16), 7595–7600 (2004). https://doi.org/10.1063/1.1794635

[27] Aidas, K., Angeli, C., Bak, K.L., Bakken, V., Bast, R., Boman, L., Christiansen, O., Cimiraglia, R., Coriani, S., Dahle, P., Dalskov, E.K., Ekström, U., Enevoldsen, T., Eriksen, J.J., Ettenhuber, P., Fernández, B., Ferrighi, L., Fliegl, H., Frediani, L., Hald, K., Halkier, A., Hättig, C.,

Heiberg, H., Helgaker, T., Hennum, A.C., Hettema, H., Hjertenæs, E., Høst, S., Høyvik, I.-M., Iozzi, M.F., Jansík, B., Jensen, H.J.A., Jonsson, D., Jørgensen, P., Kauczor, J., Kirpekar, S., Kjærgaard, T., Klopper, W., Knecht, S., Kobayashi, R., Koch, H., Kongsted, J., Krapp, A., Kristensen, K., Ligabue, A., Lutnæs, O.B., Melo, J.I., Mikkelsen, K.V., Myhre, R.H., Neiss, C., Nielsen, C.B., Norman, P., Olsen, J., Olsen, J.M.H., Osted, A., Packer, M.J., Pawlowski, F., Pedersen, T.B., Provasi, P.F., Reine, S., Rinkevicius, Z., Ruden, T.A., Ruud, K., Rybkin, V.V., Sałek, P., Samson, C.C.M., de Merás, A.S., Saue, T., Sauer, S.P.A., Schimmelpfennig, B., Sneskov, K., Steindal, A.H., Sylvester-Hvid, K.O., Taylor, P.R., Teale, A.M., Tellgren, E.I., Tew, D.P., Thorvaldsen, A.J., Thøgersen, L., Vahtras, O., Watson, M.A., Wilson, D.J.D., Ziolkowski, M., Ågren, H.: The Dalton quantum chemistry program system. WIREs Comput. Mol. Sci. **4**(3), 269–284 (2014). https://doi.org/10.1002/wcms.1172

[28] Hebling, J., Almási, G., Kozma, I.Z., Kuhl, J.: Velocity matching by pulse front tilting for large-area THz-pulse generation. Opt. Express **10**(21), 1161–1166 (2002). https://doi.org/10.1364/OE.10.001161

[29] Fülöp, J.A., Ollmann, Z., Lombosi, C., Skrobol, C., Klingebiel, S., Pálfalvi, L., Krausz, F., Karsch, S., Hebling, J.: Efficient generation of THz pulses with 0.4 mJ energy. Opt. Express **22**(17), 20155–20163 (2014). https://doi.org/10.1364/OE.22.020155

[30] Sajadi, M., Wolf, M., Kampfrath, T.: Transient birefringence of liquids induced by terahertz electric-field torque on permanent molecular dipoles. Nat. Commun. **8**(1), 14963 (2017). https://doi.org/10.1038/ncomms14963

[31] Wu, Q., Zhang, X.-C.: Free-space electro-optic sampling of terahertz beams. Appl. Phys. Lett. **67**(24), 3523–3525 (1995). https://doi.org/10.1063/1.114909

[32] Nahata, A., Weling, A.S., Heinz, T.F.: A wideband coherent terahertz spectroscopy system using optical rectification and electro-optic sampling. Appl. Phys. Lett. **69**(16), 2321–2323 (1996). https://doi.org/10.1063/1.117511

[33] Brunner, F.D.J., Johnson, J.A., Grübel, S., Ferrer, A., Johnson, S.L., Feurer, T.: Distortion-free enhancement of terahertz signals measured by electro-optic sampling. I. Theory. J. Opt. Soc. Am. B **31**(4), 904–910 (2014). https://doi.org/10.1364/JOSAB.31.000904

[34] Liptay, W.: Electrochromism and Solvatochromism. Angew. Chemie Int. Ed. English **8**(3), 177–188 (1969). https://doi.org/10.1002/anie.196901771

[35] Bublitz, G.U., Boxer, S.G.: STARK SPECTROSCOPY: Applications in Chemistry, Biology, and Materials Science. Annu. Rev. Phys. Chem. **48**(1), 213–242 (1997). https://doi.org/10.1146/annurev.physchem.48.1.213

[36] Aubret, A., Orrit, M., Kulzer, F.: Understanding Local-Field Correction Factors in the Framework of the Onsager-Böttcher Model. ChemPhysChem **20**(3), 345–355 (2019). https://doi.org/10.1002/cphc.201800923

[37] Stanley, R.J., Siddiqui, M.S.: A Stark Spectroscopic Study of N(3)-Methyl, N(10)-Isobutyl-7,8-Dimethylisoalloxazine in Nonpolar Low-Temperature Glasses: Experiment and Comparison with Calculations. J. Phys. Chem. A **105**(49), 11001–11008 (2001). https://doi.org/10.1021/jp011971j

[38] Premvardhan, L., Peteanu, L.: Electroabsorption measurements and ab initio calculations of the dipolar properties of 2-(2'-hydroxyphenyl)-benzothiazole and -benzoxazole: two photostabilizers that undergo excited-state proton transfer. Chem. Phys. Lett. **296**(5), 521–529 (1998). https://doi.org/10.1016/S0009-2614(98)01048-3

[39] Isnardi, H.: Die Dielektrizitätskonstante von Flüssigkeiten in ihrer Temperaturabhängigkeit. Zeitschrift für Phys. **9**(1), 153–179 (1922). https://doi.org/10.1007/BF01326964

[40] Rønne, C., Jensby, K., Loughnane, B.J., Fourkas, J., Nielsen, O.F., Keiding, S.R.: Temperature dependence of the dielectric function of C6H6(l) and C6H5CH3(l) measured with THz spectroscopy. J. Chem. Phys. **113**(9), 3749–3756 (2000). https://doi.org/10.1063/1.1287737
